# Atlanta Medical College

**Published:** 1866-03

**Authors:** 


### Atlanta Medical College.

The Eighth Regular Summer Course of Lectures in this Institution will be opened on the first Monday in May next, with a full corps of teachers. After four years of anxiety and toil in the discharge of professional duties in the field, in hospitals, and in private practice, five of the original faculty have "*reported for duty*" at the halls of science, where medicine and surgery for four years have been practiced, but are now taught. Two of our esteemed confreres leave us. Drs. H. W. Brown and J. W. Jones are compelled, from ill health, to sever their connection with the College. Their highly useful labors were coeval with the existence of the Institution, and while we feel warranted in saying that their places have been supplied by gentlemen every way competent and worthy, we can but feel sad in parting with those, who, in the trying hour of launching our bark upon the ocean of public opinion, stood faithfully by us in bearing "the heat and burthen of the day." May success and happiness attend them through life.

Besides the changes in the chairs of Anatomy and practice of medicine, an eighth chair—Surgical and Pathological Anatomy—which has not before been filled, is now supplied by a popular Confederate Surgeon in the recent war.

It will be seen from an announcement published in this number of the Journal that the Regular Summer Course of Lectures will be opened in the College the first of May. This will afford young men engaged in the study of medicine rare facilities for the rapid advancement in the pursuit of medical knowledge.

The great difficulty in the education of young men for the medical profession exists in the loss of time, and the loss of knowledge already obtained, during the eight months interval between the usual four months courses. The time spent in a private office is not generally so improved as to retain even till the next course what was learned at the last. And to a beginner the advantages above those found in private reading at home are equally important. . The facility with which every branch of medical science can be taught during summer has been satisfactorily demonstrated here for several years, and the location, so far as convenience, comfort and health are concerned, affords no cause of complaint.

